# Plasma Assisted Reduction of Graphene Oxide Films

**DOI:** 10.3390/nano11020382

**Published:** 2021-02-03

**Authors:** Sri Hari Bharath Vinoth Kumar, Ruslan Muydinov, Bernd Szyszka

**Affiliations:** Institute of High-Frequency and Semiconductor System Technologies, Technische Universität Berlin, HFT 5-2, Einsteinufer 25, 10587 Berlin, Germany; ruslan.muydinov@tu-berlin.de (R.M.); bernd.szyszka@tu-berlin.de (B.S.)

**Keywords:** graphene oxide, plasma treatment, reduction

## Abstract

The past decade has seen enormous efforts in the investigation and development of reduced graphene oxide (GO) and its applications. Reduced graphene oxide (rGO) derived from GO is known to have relatively inferior electronic characteristics when compared to pristine graphene. Yet, it has its significance attributed to high-yield production from inexpensive graphite, ease of fabrication with solution processing, and thus a high potential for large-scale applications and commercialization. Amongst several available approaches for GO reduction, the mature use of plasma technologies is noteworthy. Plasma technologies credited with unique merits are well established in the field of nanotechnology and find applications across several fields. The use of plasma techniques for GO development could speed up the pathway to commercialization. In this report, we review the state-of-the-art status of plasma techniques used for the reduction of GO-films. The strength of various techniques is highlighted with a summary of the main findings in the literature. An analysis is included through the prism of chemistry and plasma physics.

## 1. Introduction

**Figure 1:**
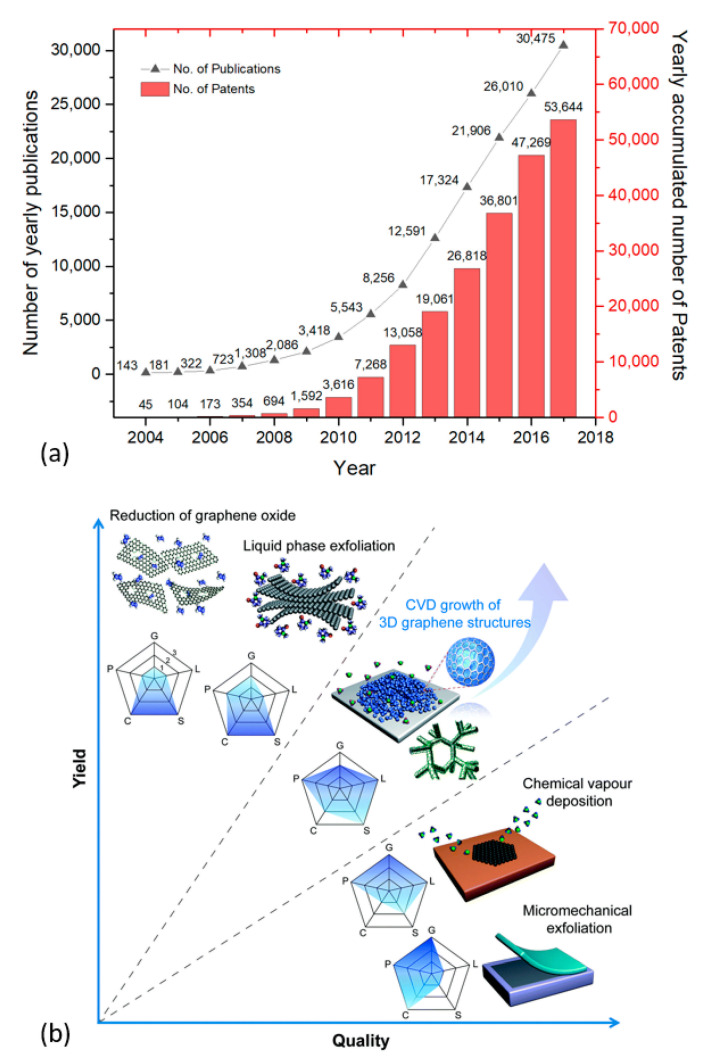
(**a**) The yearly number of publications related to graphene topic (left-side axis). Data extracted from the Web of Science^TM^ (Clarivate Analytics), searched with the “graphene” keyword in the “topic” field (date of retrieval: September 14, 2020). Yearly accumulated graphene-related patents (right-side axis), data extracted from [29]. (**b**) Schematic illustration of some of the main graphene production techniques represented in terms of yield and quality. The evaluation of the technique’s production process is represented in a pentagon with graphene crystallinity (G), purity (P), layer number controllability (L), cost (C, a low value is related to the high cost of production), and scalability (S). Numbers 1, 2, and 3 indicate low, medium, and high, respectively. Reproduced from Chen et al. [30] with permission from The Royal Society of Chemistry.

The term “graphene” was coined by Boehm et al. in 1985, which refers to a two-dimensional single layer of carbon atoms in a honeycomb lattice [1]. A. Geim and K. Novoselov exfoliated graphene for the first time in the year 2004, which consequently earned them a Physics Nobel prize in 2010. Even before its discovery and eventually gaining the “wonder material” nickname [2], graphene was known to scientists and used in theoretical studies dating back to 1947 [3,4,5,6,7,8,9]. Following the discovery, graphene has gained a lot of attention from the scientific community across various disciplines (Figure 1a). This can be credited to its remarkable electrical, optical, thermal, and mechanical properties [9,10,11]. Additionally, it possesses complete non-permeability to all standard gases [12] and the ability to be chemically functionalized [13,14]. 

Figure 1b presents schematic illustrations of the common production methods of graphene. A detailed account of various production and processing techniques of graphene and related materials can be found in the literature [15,16,17,18]. Methods such as mechanical exfoliation [19,20], epitaxial synthesis [20,21], and bottom-up synthesis from structurally defined organic precursors [20] restrict the use of graphene to fundamental research and niche applications, owing to limited scalability and high production costs. Graphene layers can be also obtained by chemical vapor deposition (CVD), a well-established technique in the industry [22,23]. The downside to this technique is that it requires suitable substrates (which are limited), a high temperature, and vacuum environment. Additionally, it involves the laborious transfer of the grown layers onto desired application substrates [23]. In liquid-phase exfoliation (LPE), pristine or expanded graphite particles (thermally expanded graphite intercalation compounds) are first dispersed in a solvent to weaken van der Waals attraction between the graphene layers. High-quality graphene sheets are then obtained by following ultrasonication [24], electric field [25], shearing [26], and microfluidization [27] to induce exfoliation of graphite layers. Chemical additives (surfactants) are often needed to keep the suspensions stable for a long period, and the removal of solvent may cause restacking of the graphene platelets due to van der Waal’s forces [17]. Some solvents, such as the N-methyl-pyrrolidone (NMP), are toxic and expensive with a high boiling point mandating special treatment and handling [28]. 

Compared to other techniques, the reduction techniques of GO yield relatively lower product quality but provide some interesting characteristics. GO itself is highly hydrophilic and can form stable monolayers in aqueous colloids [31,32]. GO and rGO can be chemically functionalized through covalent and non-covalent bonds to enhance their properties and functionalities. In the non-covalent approach, they can be modified with metals, metal oxides, and polymers through non-covalent interactions like van der Waal’s forces, π–π stacking, hydrogen bonding, hydrophobic interactions, and ionic crosslinking [33]. In applications such as sensors, energy storage, electrochemical systems, catalysis, etc., the superior properties arise from the reactivity of the intrinsic defects and dangling bonds in GO [34]. 

Numerous review-articles on synthesis, structure and properties, fabrication techniques, chemical modifications, and applications of GO are available [13,16,35,36,37,38,39,40,41]. On the reduction front, specific methods (such as chemical, thermal, eco-friendly, microwave methods, etc.), as well as an overview of several reduction approaches have been examined [42,43,44,45,46,47,48,49,50,51,52,53,54]. Amongst these, some reviews have only outlined the plasma method along with other techniques [49,51]. The use of plasma for GO modification and functionalization also has been reported [14,55]. This article aims to review the application of plasma exclusively from the perspective of GO reduction, presenting an up-to-date analysis. The primary objective here is to elucidate the reduction of GO-films with various plasma and to highlight the potential of plasma technologies in this topic. GO in the form of monolayers, thin-films, and paper (a few μm-thick interlocked layered-structure consisting of micrometer-sized graphene crystals [56,57]) is covered here apart from GO-composites [58] and powders [59,60]. The fundamentals, principles, configurations, and applications of plasma are not covered, as they are available in literatures [61,62,63,64,65,66,67]. A table is included in the Appendix A (Table A1) with a non-exhaustive list of relevant publications for easy referencing. It incorporates various plasma generation techniques and active gases employed, briefing the important experimental parameters, application, and results.

## 2. GO/rGO: Properties, Reduction Methods, and Characterization

In this chapter, the structure, properties, and applications of GO/rGO are briefly discussed first. It is then followed by a short overview of common reduction techniques highlighting the advantages of plasma methods. Finally, the basic characterization techniques needed to evaluate the reduction degree in rGO-films are introduced.

### 2.1. Structure and Properties of GO/rGO

GO is strictly a single-layered material that is obtained by exfoliation of oxidized graphite [68]. Oxidized graphite depicts a berthollide layered solid produced by treating graphite with strong oxidants where the graphite surface and edges undergo covalent chemical oxidation [68]. This technique dates back to 1859 when the chemist Benjamin Brodie performed a similar treatment to elucidate the structure of graphite oxide [69]. As a result of chemical exfoliation, GO incorporates many oxygen-containing functional groups where domains of sp^2^- and sp^3^-hybridized carbon atoms exist [70]. 

**Figure 2:**
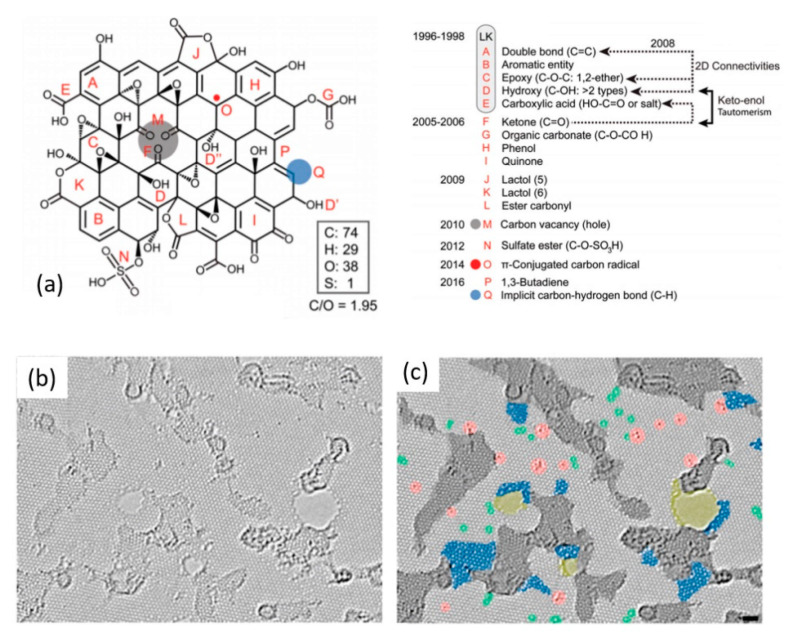
(**a**) A historical structural account of a GO-flake with C/O atomic ratio of ~2. Reproduced from Brisebois et al. [76] with permission from The Royal Society of Chemistry. The atomic resolution, aberration-corrected TEM image of an H_2_-plasma reduced monolayer GO: (**b**) Original image and (**c**) with colors highlighting different features. Light gray: defect-free crystalline graphene area. Dark gray: contaminated regions. Blue: disordered single-layer carbon network, or extended topological defects, suggested as remnants of the oxidation-reduction process. Red: individual ad-atoms or substitutions. Green: isolated topological defects, that is, single bond rotations or dislocations cores. Yellow: holes and their edge reconstructions. Reprinted with permission from Gomez-Navarro et al. [82]. Copyright © 2010, American Chemical Society.

Until today, some ambiguity persists on the precise chemical structure of GO, and several different models describe the same [52,71,72]. The most widely accepted model is the one proposed in 1996 by Lerf and Klinowski (LK) [73,74], originally describing graphite oxide. In the past two decades, various researchers have claimed additional structural changes [75,76]. In a recent review, Brisebois et al. [76] presented a representative structure of GO based on historic and modern models including recently suggested adjustments in the literature (Figure 2a). As shown in Figure 2a, features A–E account for the LK model. The monolayer surface gets its nearly flat carbon-grid from double bonds (A), aromatic entities (B), and epoxide groups (C). Hydroxyl group-containing carbon results in wrinkling of the monolayer. A large number of oxygen-containing groups (C, D, D’, and D”) lie above and below the carbon-grid. Hydroxyl groups (D’) and carboxylic groups (E) terminate the structure of GO. Features such as F, G, H, and I are aspects of the Dékány model [76]. According to Brisebois et al. [76], the complete chemistry of GO is not yet fully understood, and the general LK model should be updated with recently made observations such as carbon vacancies (M), sulfate esters (N), carbon radicals (O), 1,3 butadiene systems (P), and C–H bonds (Q). 

The mainly present oxygen-functional groups in GO are epoxides (C-O-C), hydroxyls (-OH), carboxylic (-COOH), and ketone (C=O) ones [77]. The presence of polar groups makes GO hydrophilic and facilitates its exfoliation in aqueous media [78]. The incorporated functional groups cause an increased interlayer distance of >0.625 nm in GO from 0.335 nm in graphite [79,80]. GO has a heterogeneous chemical and electronic structure, and the presence of oxygen-containing groups make it an insulator [35,81]. The sp^2^ network of carbon atoms, and thus the electrical conductivity, can be substantially restored by various reduction strategies. Figure 2b presents high-resolution imaging of rGO monolayer reduced with H_2_-plasma [82]. A large portion of crystallized graphene regions with hexagonal lattice (light gray) is observed. The average graphene-like regions here range from 3–6 nm covering 60% of the surface. Carbonaceous adsorbates and trapped contaminants are also observed (dark gray). Other visible features include topological defects (blue and green), individual ad-atoms or substitutions (red), and holes (yellow). Such heterogeneous structure gives rise to properties that are different from pristine graphene. Some of the important properties of GO/rGO are summarized against CVD-graphene in Table 1.

The opportunity for tailoring the optoelectronic properties of GO arises from the ability to manipulate its shape, size, and the fraction of sp^2^/sp^3^ hybridized carbon domains by controlled reduction [35,106]. Finally, GO and r-GO serve as a tunable platform for several applications. Table 2 summarizes some of the features and properties of GO/rGO exploited in a wide range of science and technology topics. In optoelectronics, under the field of solar-cells alone, rGO has contributed to the progress of perovskite, perovskite-silicon tandem, dye-sensitized, and organic technologies [107,108,109]. Graphene materials have not been widely utilized for CIGSe as in the case of other solar cell technologies. Nevertheless, CVD–graphene has been incorporated into CIGSe, which includes a demonstration of flexible solar cell [110,111,112]. It is emphasized that GO/rGO can serve as hole-selective contacts and intermediate tunnel junction layer in monolithic CIGSe–Perovskite tandem solar cell applications, which is yet to be reported [113]. Factors such as precursor material-form (powder, dispersion, films, paper, etc.), quality, properties, and application of rGO can influence the choice of reduction method adopted, which is discussed in the following section.

### 2.2. Reduction Methods for GO

Table 3 lists and summarizes commonly employed reduction methods and some of their important features along with those of the plasma method. Generally, the reduction strategies use either a reductant (chemical, microbial, solvothermal) and/or a thermal, electrical (voltage-induced), radiative (photocatalytic, microwave, plasma), and electrochemical impact [49,119]. Chemical reagents are either used in liquid- or gas-phase for reduction [32,120]. Typically used reagents are fairly hazardous: hydrazine [94], hydrazine hydrate [121], sodium borohydride [122], sodium hydrosulfite [122], and hydrohalic acids [121]. Hydrazine is known to be one of the most powerful reducing agents. However, high toxicity and environmental hazard make it unpopular [123]. By comparing various reducing agents, a study suggested that a reducing agent in combination with acid is beneficial in terms of preserving the rGO surface quality [124]. The processing time in the chemical techniques is relatively longer. In certain cases, the reduction process can consume a day to a week’s time [94,120]. 

The solvothermal method can yield a stable dispersion of r-GO without the use of additional reductants [131]. However, the C/O atomic ratio and electrical conductivity obtained are inferior to the chemical methods [42,52]. Microbes such as *Shewanella* [132]*, E. coli* [133]*,* yeast [134]*,* and *Azotobacter chroococcum* [135] can also reduce GO in the forms of dispersion or film. Though such biological agents are attractive with low-negative environmental impact, they are limited in terms of the need for sensitive culture procedure and prolonged reaction time [49]. 

High-temperature annealing (up to 1100 °C) aids the removal of oxygenated groups and significantly improves the conductivity of GO-films [136]. This approach is not applicable for temperature-sensitive substrates, like glass or flexible ones. When the preheating and cooling of chambers with active/inert gases or vacuum environment are considered, a substantial amount of energy is consumed, leading to poor energy efficiency. Another significant drawback of the thermal method is the creation of carbon vacancies and other structural defects in the GO plane due to the active diffusion of epoxide groups already at 200 °C [137,138]. Thermogravimetric analysis reveals up to ~70% final mass loss caused by the release of CO and CO_2_ gases [137]. The photocatalytic reduction of GO heavily relies on the presence of photoactive materials under UV radiation. This makes it suitable only for hybrid nanocomposites [46]. Electrochemical reduction offers a faster, and safer route compared to the previously noticed methods. However, it is not viable for large-scale production, and its degree of reduction is incomparably lower than in the case of the chemical or thermal method [139]. 

Microwave-assisted reduction of GO can be realized by three routes: (i) chemical reduction, (ii) thermal reduction, and (iii) simultaneous exfoliation and reduction [127]. Microwaves are effectively absorbed by π-electrons that cause very rapid warming of GO (several hundreds of degrees in few seconds) that results in the breaking of weakest bonds [127]. According to this principle, r-GO domains with sp^2^ carbon network and free π-electrons heat faster than GO-ones that minimize the efficiency of this method [128,129]. When chemically reduced or simultaneously exfoliated and reduced, the quality of r-GO is low and oxygen content is as high as in the case of the conventional thermal method [54,127]. Laser irradiation can also induce local heating in light-absorbing domains of GO and thus be utilized for reduction. The local temperatures can reach 1400 K in this case [140]. In the case of GO films the heat absorbed dissipates to underlying layers and substrate, yielding additional issues. The photothermal reduction (such as the laser and flash techniques) can provide moderate to high atomic C/O ratio (~10 and ~15, respectively) but creates pores, cracks, and voids in GO-films, which could limit its applications [141]. 

Compared to the previously discussed methods, plasma-assisted reduction techniques are much more attractive for films due to the following reasons. Plasma processes are established and well-controlled, which offers ease of operation also on the industrial scale. Despite relatively expensive equipment, the versatility of plasma processes today forms a vital part of production in various technological fields [67]. Different power generation techniques extended by a wide range of operating pressures including the atmospheric one, as well as the applicability of various active gases, make this approach multidimensional. It opens the way to control the energy of the acting species and tune the chemical footprint of plasma. Moreover, the thermal impact of plasma and the depth of penetrating damage can be also restricted. For attaining graphene-like properties in rGO, significant restoration of the graphitic structure by defect repair is essential. Amongst the available techniques to realize this, the plasma method is one of them, others being the thermally assisted CVD method (>1073 K) and sequential chemical reduction followed by high-temperature graphitization (~2073 K) [48,142]. From environmental, health, and safety aspects, plasma technology has remarkable advantages over chemical and thermal processes [143]. Owing to their advantages, plasmas have demonstrated their attractiveness in the synthesis of graphene and related materials [130,144,145,146].

### 2.3. Characterization of rGO

The effectiveness of GO reduction is widely assessed based on (i) surface atomic carbon/oxygen ratio (R_C/O_) and (ii) electrical properties: hole/electron mobility (μ), sheet resistance (R_SH_), and conductivity (σ). Detailed reports on various GO characterization techniques generally utilized are available in the literature [18,147,148]. When comparing the electrical parameters of various r-GO films, one should consider that monolayers, bi-layers, and tri-layers of r-GO may not differ proportionally. For instance, according to Sinitskii et al. [149], the corresponding conductivities of reduced GO-nanoribbons were found to be 35, 115, and 210 S/cm. In certain cases, such a difference between mono-layers and bi-layers was attributed to the interaction between r-GO and the underlying substrate [150,151]. The R_C/O_ values determined by a surface-sensitive X-ray Photoelectron Spectroscopy (XPS) indicate a degree of reduction. In the case of the layers thicker than 10 nm, XPS is unable to validate the reduction degree in the bulk [152,153]. 

It is worth noticing characterization of graphene films by Raman spectroscopy, as it provides vital insights. Graphitic materials have Raman features at ~1584 cm^−1^ (G-band), ~2700 cm^−1^ (G’-or 2D-band), and ~1350 cm^−1^ (D-band). The G-band arises from the first-order scattering of E_2g_ phonons of the sp^2^ carbon atoms in the ring structure, while the D-band appears from the breathing mode of sp^2^ carbon atoms due to defects [154,155]. The G’-band, unlike the D-band, is not induced by defects and is more prominent in graphene [155]. In graphene, the integrated intensity ratio of G’- and G-band is used to determine the number of layers [22,156]. The G’-band intensity declines, and its full width at half maximum (FWHM) broadens with increasing density of defects [157]. The integrated intensity ratio of D-band and G-band (I_D_/I_G_) is widely used for characterizing the defects’ quantity in graphene and related materials. The Tuinstra–Koenig empirical relation [158] based on the I_D_/I_G_ is used to calculate the in-plane sp^2^ carbon crystallite size (L_a_)_._ The average distance between point defects (L_D_) can also be derived from the I_D_/I_G_ value and the FWHM of the G-band [124,157,159]. Wróblewska et al. [160] highlighted the difficulty in comparison of materials with widely varied I_D_/I_G_ ratios reported in the literature. For instance, inhomogeneity of GO/r-GO may cause a difference in values measured at distances of a dozen of μm. Additionally, the ratio in question also depends on the laser wavelength used [160]. To reduce uncertainty, a statistical approach (Raman mapping) instead of using single-point measurement should be taken [124,160]. This is unfortunately not the case in every investigation.

Fourier-transform infrared spectroscopy (FTIR) is also a useful tool to investigate the effectiveness of GO-reduction. The presence of various oxygen-containing groups can be recognized in GO/rGO, and thus their removal can be examined [161,162]. The configurations that can be identified with FTIR are [163]:

- epoxide (C-O-C): 1230–1320 cm^−1^, asymmetric stretching; ~850 cm^−1^ bending motion,
- sp^2^-hybridized C=C: 1500–1600 cm^−1^, in-plane vibrations,
- carboxyl (COOH): 1650–1750 cm^−1^ (including C-OH vibrations at 3530 and 1080 cm^−1^),
- ketonic species (C=O): 1600–1650 and 1750–1850cm^−1^_,_ and
- hydroxyl (namely phenol, C-OH): 3050–3800 and 1070 cm^−1^) with all C-OH vibrations from COOH and H_2_O.

## 3. Plasma-Assisted Reduction of GO

**Figure 3:**
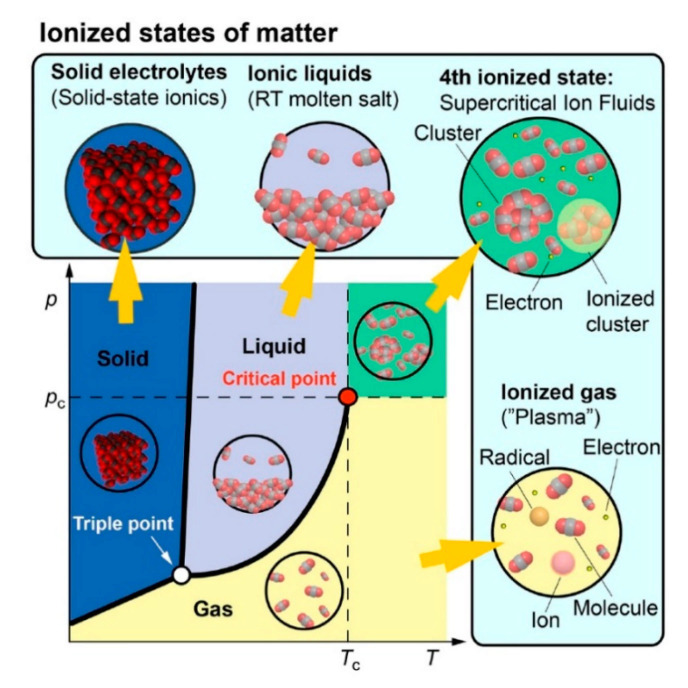
Schematic of a typical phase diagram depicting corresponding ionized states of matter. Reproduced from Adamovich et al. [67]. Copyright © 2017 IOP Publishing Ltd, licensed under CC BY 3.0.

Chemist Irving Langmuir coined the term “plasma” in 1928 [164], which often denotes the fourth state of matter (see Figure 3). In the visible universe, more than 99% of constituents are expected to be in a plasma state (center of active stars, corona flares and sunspots, magnetospheres of the earth, comet-tails, inter-stellar and inter-galactic media, and in the accretion disks around black holes) as opposed to the condensed matter (solids, liquids, and gases) in the form of comets, planets, and cold stars [165,166,167]. Plasma is the ionized form of gases containing energetic ions, free electrons, highly reactive radicals, and photons. The extent of ionization can range from very low values (ionized fraction in the order of 10^−4^–10^−6^) up to full ionization [65]. If all species in plasma have the same temperature (or energy), one deals with an equilibrium plasma state. In non-equilibrium plasmas, electrons have higher temperatures than the remaining species. Besides that, laboratory plasma can be divided into high-temperature plasma (or fusion plasma, e.g., in tokamaks, z-pinch system, etc.) and low-temperature plasma (or gas discharge) [65]. The latter is relevant for us, and according to Szabó et al. [168], it can be further classified as follows:

- operating pressure:
  o low-pressure plasma
  o atmospheric pressure plasma
- temperature:
  o low-temperature plasma (T_gas_ < 2000 K)
  o high-temperature plasma (T_gas_ > 2000 K)
- thermodynamics:
  o thermal plasma/equilibrium plasma (T_electron_ ≈ T_ion_ ≈ T_gas_)
  o non-thermal plasma/non-equilibrium plasma (T_electron_ ≫ T_ion_ ≈ T_gas_)
- type of coupling:
  o inductive coupling
  o capacitive coupling
- plasma generation:
  o microwave discharge (300 MHz ≤ f ≤ 300 GHz)
  o radiofrequency (RF) discharge (ideally 13.56 MHz):
  o direct current (DC) discharge
  o dielectric barrier discharge (DBD)
  o corona discharge
  o electric arc
  o hollow cathode discharge
  o electron beam discharge (EB)
  o plasma torch
  o alternating current.

Based on the operating pressure, the low-temperature plasmas can be broadly classified into low pressure and atmospheric pressure plasma. Traditional sources of atmospheric plasma include the transferred arcs, plasma torches, corona discharges, dielectric barrier discharges (DBD), and atmospheric-pressure plasma jet (APPJ) [63]. The classical arc torches (ones with local thermal equilibrium) are characterized with high gas temperatures and have been used in applications such as welding, cutting, spraying, etc., where heat is required [169]. Relatively modern low-powered homogeneous arc plasma, generating less heat, is well implanted in the production lines of automobiles, textiles, and packaging, etc. [169]. Corona discharges are spatially non-uniform and are formed on sharp-points, edges, or on thin-wires where the electric field is very large [170]. As the active volume is limited, they are not well suited for the industrial production of large quantities of chemical species [61].

The DBD was developed to overcome disadvantages of the corona discharge [169]. Amongst the atmospheric plasmas, the DBD is better suited for the applications needing volume plasma chemistry, as it caters to large volume excitation with energetic electrons for excitation of atomic and molecular species breaking chemical bonds [61]. The APPJ plasma shares a similar plasma density as the low-pressure plasma but with lower breakdown voltage and electron temperatures than the rest of the plasmas. However, the population of electrons is considered high enough to dissociate many molecules including O_2_ and N_2_ [63]. The main disadvantage of the APPJs is the small area that can be treated or coated, which can be circumvented with approaches such as scanning of surface area, using an array of APPJs, and rotating arc root plasma jet process [171]. In a recent review [14], the APPJ-plasma was emphasized to be a promising candidate for large-scale roll-to-roll functionalization of graphene and GO.

The low-pressure plasma emerges from the field of material processing and is a key player in the semiconductor industry [172,173]. Low-pressure plasma treatment of electronic devices, printed circuit boards, and semiconductors are state of the art. Uniform treatment of oxidation-sensitive and three-dimensional objects can be carried out, including cavities that can be processed in large chambers (up to 12,000 L in volume) [174]. They feature some distinctive benefits: (1) uniform glow over large areas, (2) high concentration of reactive species (able to etch or deposit at the rate of up to 10 μm/min), (3) lower breakdown voltages, (4) stable operating window, and (5) sufficient electron temperatures to dissociate molecules with lower gas temperature [63]. On the downside, the vacuum systems are relatively expensive in assembly and maintenance. Furthermore, the processing is limited by batching and transferring materials in and out of the vacuum system. Some of the plasma parameters of low-pressure and atmospheric-pressure plasma are summarized in Table 4.

The plasma-assisted GO-reduction can be regarded analogically to the plasma etching process in a way [66]. It should selectively remove oxygen-containing groups, leaving the carbon network unaffected. On a solid surface exposed to the plasma, two processes track simultaneously: (i) deposition of material and (ii) ablation leading to its removal. Both are determined by the discharge gas and conditions [175]. The ablation of the treated surface can involve sputtering, chemical etching, ion-enhanced energetic etching, and ion-enhanced protective etching [66,175]. In the case of the plasma-assisted chemical etching, the plasma species are excited and become chemically more reactive. In case of the ion-induced etching, plasma activates surface atoms that increase their ability to release under certain pressure and chemical conditions [176,177]. Admixing the inhibitor species into the gas phase results in an isotropic inert coverage of the treated surface, preventing further etching. In sputtering, a bombardment of the surface by the ions with sufficient kinetic energy can break chemical bonds in the solid and eject atoms into a gas phase. Plasma process is quite complex and dynamic where several processes occur simultaneously being subjected to the plasma generation conditions. 

The first use of a plasma process for GO reduction was reported in the year 2007 [150], two years following the first report on GO solution processing [31]. Since then, in the last 13 years, several kinds of plasma-assisted reduction processes were developed. To name some ways plasma generation is utilized: radio-frequency (RF) plasma [150,162,178,179,180,181,182,183,184,185,186,187,188,189,190,191,192,193,194,195,196,197], low-pressure direct current (DC) plasma [198,199,200,201], micro-DC plasma [202], atmospheric pressure glow discharge (AGD) plasma [56], electron beam (EB) plasma [203], active screen (AS) plasma [204], atmospheric pressure plasma jet (APPJ) [205] and μ-APPJ [206], and dielectric barrier discharge (DBD) plasma [207,208]. In the following sections, the reduction processes classified according to the discharge gas will be discussed in detail.

### 3.1. Inert-Gas (He and Ar) Plasma

**Figure 4:**
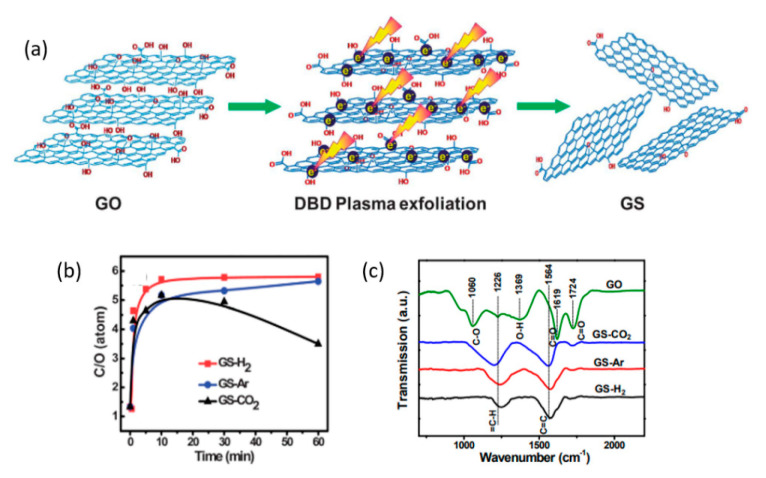
(**a**) A schematic representation of the DBD plasma exfoliation of GO. (**b**) R_C/O_ as a function of treatment time with from H_2_, Ar, and CO_2_ DBD plasma. (**c**) FTIR spectra of GO and graphene prepared by DBD plasma with different type of working gases. Reproduced from Zhou et al. [209] with permission from the Royal Society of Chemistry.

Zhou et al. [209] used a 60 W (AC) DBD plasma with several discharge gases (Ar, H_2_, and CO_2_) for simultaneous exfoliation and reduction of GO powder. The plasma treatment mechanism suggested dictates similar for the GO-films. According to this work, the alternating electric field distorts polar bonds in oxygen-containing groups. Furthermore, high-energy electrons and ions of plasma bombard GO surface, rupturing the bonds of oxygen-containing groups within nanoseconds. Jin et al. [210] have shown by first-principle calculations that provision of an electron to the hydroxyl group favors its desorption from the GO surface. The plasmas in general have a high density of electrons (see Table 3); therefore, desorption of hydroxyl groups must be a frequent event. Zhou et al. demonstrated that the deoxygenation of GO was strongly influenced by the discharge gas (see Figure 4). When Ar was employed, deoxygenation occurred primarily through the bombardment of energetic ions and electrons, unlike the H_2_-plasma, which could also provide chemically reactive plasma species (H, H^+^, H_2_^+^, and H_3_^+^ [211]). Other authors have reported that a combination of inert gases and a reactive gas can be more effective in reduction compared to pure inert or reactive gas plasma; this will be discussed in the following sections.

Cardinali et al. [188] used a 25 W RF plasma with Ar for simultaneous thinning and reduction of the bulk GO platelets. Starting from the thickness of ~600 nm, the authors etched samples for 40 min down to 5–6 nm thick multi-layered film with over two orders lower surface electrical resistivity. Although an inert gas plasma does not provide chemically reactive species that can act as reductant, the bombardment by energetic inert ions and electrons have proven to be adequate for deoxygenation in some reports. Bo et al. [200] used an instantaneous 2-s atmospheric-pressure glow discharge process with helium for preparing rGO-paper. The plasma-treated rGO-paper (σ: 59 S/cm; R_C/O_: 7.6) was on par with the one reduced chemically by hydrazine hydrate (σ: 65 S/cm; R_C/O_: 8.5). The authors attributed instantaneous deoxygenation with their plasma to the synergy of high-density electrons and heating. The electron density and the neutral gas temperature were determined to be 1.03 × 10^16^ cm^−3^ and ~800 K, respectively. Herewith, no damage to the graphitic structure was found by Raman spectroscopy. The r-GO layers reduced by inert-gas plasma have been demonstrated in supercapacitors [200] and H_2_O_2_ chemical sensors [162].

**Figure 5:**
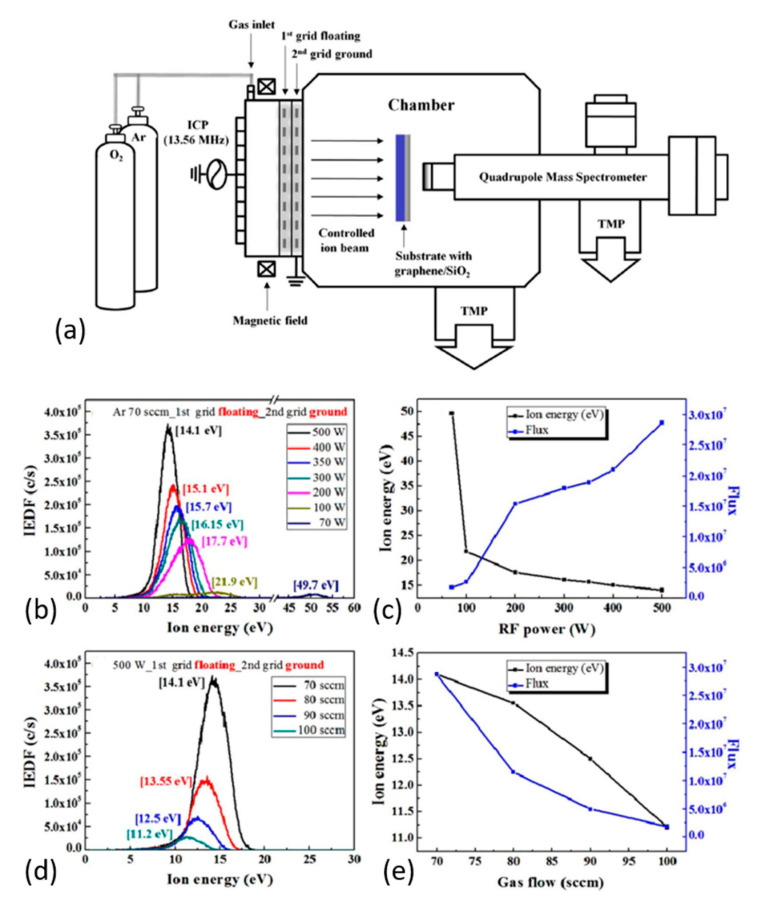
(**a**) Schematic of a two-grid ICP-type ion beam system with axial magnetic field used for atomic layer etching of graphene with a quadrupole mass spectrometer (QMS, for ion energy/flux measurement of the ion beam). (**b**) Various RF powers at 70 sccm of Ar gas flow rate and (**c**) the corresponding peak energies and flux for their Ar^+^-ion energy distributions. (**d**) Various Ar flow rates at 500 W RF power measured by an ion energy analyzer in the QMS and (**e**) are the corresponding peak energies and fluxes for their Ar^+^-ion energy distributions. Reproduced from Kim et al. [212]. Copyright © 2017 Authors, licensed under CC BY 4.0.

Kim et al. [212] accomplished selectively etching an atomic-layer of graphene without damaging the underneath layers using an inductively-coupled plasma-type ion beam system (see Figure 5a). The cyclic etching process used consisted of chemical adsorption of low-energy oxygen-ions: O_2_^+^ and O^+^ (0–20 eV) followed by physical desorption of oxidized species by Ar^+^-ions (11.2 eV). To control the energy of ions, the authors applied floated and grounded grids with an axial magnetic field. This approach helped to optimize the process based on ion energy distribution for various power and gas-flows in the system (see Figure 5b–e). This cyclic etch-process exploited the fact that the binding energy of the surface C-atoms decreases from ~6.1 to ~3.9 eV with chemisorption of oxygen ions. At the same time, the C–C binding energy in the underneath layer remains almost unchanged (~0.1 eV). Exactly this fact in combination with controlled energy of Ar^+^-ions has allowed etching of the top layer selectively. This experience opens the way to combine the chemical and energetic impacts of plasma species for effective and controlled GO reduction. 

### 3.2. Hydrogen Plasma

A hydrogen gas discharge can constitute free electrons, neutrals (molecular H_2_, atomic H), and charged ions (H^+^, H^−^, H_2_^+^, H_3_^+^) interacting through a set of numerous reactions [213]. A bombardment by H, H^+^, H_2_^+^, and H_3_^+^ species with energies varying from 10 eV to few hundred of eV is known to result in the etching of graphene [214,215,216]. The impact of hydrogen plasma on GO is similar to the etching effect and removal of oxygen-containing groups [193,194,208]. A molecular dynamics study [215] on graphene suggested that surface reaction strongly varies with incident atomic H energy: (i) atomic H with few tenths of eV can adsorb on the basal plane of surface-clean graphene, (ii) H energies 0.025–0.3 can selectively etch edges without damaging the basal plane, (iii) H energies in the range 0.3–10 eV hydrogenates basal plane without irreversibly damaging graphene (hydrogenation is reversible), (iv) H energies in the range 10–100 eV are suitable for patterning multi-layer graphene, and (iv) 10 eV H^+^ ions can etch graphene vertically and the hydrogen plasma containing more molecular (H_2_^+^ and H_3_^+^) than atomic (H^+^) ions may induce less subsurface damage in multi-layered graphene. Such detailed studies are yet to be reported for GO materials. 

**Figure 6:**
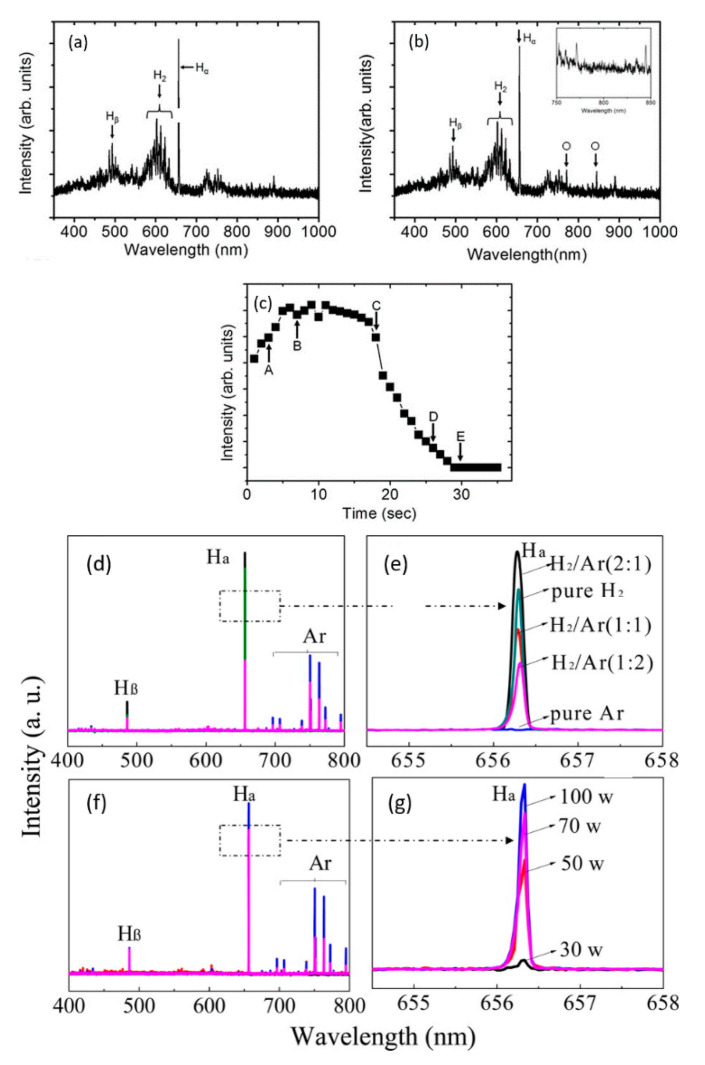
Emission spectra of H_2_ discharge without GO (**a**) and with GO sample (**b**) in the plasma treatment chamber, indicating the active species. (**c**) The OES signal from the 844.6 nm oxide line (shown in (**b**)) during the reduction of GO. A–E in (**c**) are various process points where the rGO sample properties were investigated in literature [193]. Reproduced from Kim et al. [193]. Copyright © 2013 Authors, licensed under CC BY 3.0. Emission spectra of H_2_/Ar plasma as a function of the ratio of H_2_ to Ar (**d**,**e**) and discharge power (**f**,**g**). Lines corresponding to Ar excited states and atomic hydrogen (H_α_ and H_β_) are indicated. Reprinted with permission from Li et al. [190]. Copyright © 2014 American Chemical Society.

Kim et al. [193] applied optical emission spectroscopy (OES) to determine the optimum process point for GO-reduction to avoid the degradation of electrical characteristics with excess plasma exposure. The emission corresponding to the oxygen radicals released from GO was used as an indicator of the reduction progress (see Figure 6a–c). As observed in Figure 6c, at point C (~18 s after the start of the reduction process), the intensity of the OES oxygen-line begins to decline, indicating the end of reduction. A crucial application of the OES lies in the determination of the excited states of species in the plasma. Li et al. [190] studied with this method the effect of variable plasma power and different gas mixtures (Ar/H_2_) on the reduction of GO. The emission corresponding to atomic hydrogen (in OES spectra, Figure 6d–g) was found to increase with increasing discharge power and reached an overall maximum for the H_2_/Ar ratio 2:1. The inclusion of Ar assisted in the enhanced dissociation of H_2_ due to the penning ionization. The GO (R_C/O_: 1.1) on reduction with a pure Ar and H_2_ plasma yielded rGO with R_C/O_ of 1.2 and 1.7, respectively. However, the rGO obtained with a more populous H_2_/Ar plasma (2:1) resulted in a R_C/O_ up to 6.9. Furthermore, the electrochemical performance of the fabricated rGO was demonstrated as an electrode (in KOH aqueous electrolyte), achieving a specific capacitance of 185.2 F/g. The performance was higher than several graphene-based electrodes in literature. 

### 3.3. Methane Plasma

To obtain graphene-like quality with GO as precursors, numerous investigations into reduction with healing (or repair) of defects has been carried out. By incorporating C-atoms into structural defects of GO, the sp^2^-hybridized graphene domains are restored. Various strategies employed include thermal-CVD [92,217,218,219] and high-temperature graphitization [142,220]. In a recent review, De Silva et al. [48] addressed the defect repair of GO mostly with thermal methods. This section is devoted to the plasma-enhanced CVD approach, which is more attractive in terms of large-scale applications than the others mentioned.

Methane plasma is a popular choice for the defect repair and reduction of GO. Pure discharge of CH_4_ [93], as well as in combination with Ar [180,185,203] or H_2_ [179,184,189,207,221], has been utilized. Cheng et al. [93] treated GO-monolayers on Si/SiO_2_ substrates with CH_4_-plasma (100 W, RF, ~575 °C, 10 min) that resulted in a decrease of the I_D_/I_G_ ratio from 1.03 to 0.53, indicating healing of defects and increasing conversion of sp^2^ C-atoms. They reported one of the highest conductivities (1590 S/cm) for rGO. Baraket et al. [203] used plasma generated in a CH_4_ (0–20%)/Ar gas mixture by electron-beam, resulting in the energy of electrons and dissociated ions <0.5 eV and <3 eV, respectively. By varying plasma duty factor, methane concentration, gas pressure, and treatment time, they showed that oxygen concentration in rGO can be controlled in the range 5–43 at.%. Correlating Raman, XPS, and AFM investigations, the authors concluded that the defects originating from deoxygenation were healed by the CH_n_ species. Thus, amorphous carbon was selectively rather than uniformly deposited on rGO. In a mixed gas discharge, the compositional ratio of gases is one of the important parameters to optimize. Yang et al. studied the effect of RF plasma in CH_4_/Ar mixture (100 W, no substrate heating) on GO-monolayers. They determined that the methane-rich environment (CH_4/_Ar—2:1) was the most effective for reduction.

Using the CH_4_ + H_2_ plasma can be beneficial for the following reasons: (i) the amount of reactive species is surplus for reduction of oxygen-functional groups compared to a pure CH_4_ plasma [221]; (ii) many defects and distortions of a graphitic network are bound and eliminated by the hydrogen active species and serve at once as active sites for carbon species from plasma to react [179,184], and (iii) the etching nature of H_2_ plasma restricts deposition of sp^3^ amorphous carbon from radicals of dissociated CH_4_ molecules [179,189]. The substrate temperature, CH_4_/H_2_ ratio, other plasma conditions, and treatment runtime sensitively influence the relative rates of etching and carbonization (defect restoration). Bodik et al. [207] used an instantaneous DBSCD plasma reduction (100 W/cm^3^ equimolecular CH_4_/H_2_ ratio, atmospheric pressure, 5 s) of the unheated GO films, and observed that this treatment was effective in the removal of oxygen-functional groups but not in sp^2^-C restoration. Nevertheless, such a plasma process can promote application on temperature-sensitive flexible substrates. Chiang et al. [221] performed a reduction of GO-nanoribbons films with an RF CH_4_/H_2_ plasma at 230 °C. Impressively, even at this moderate temperature, the removal of all kinds of oxygen-containing groups (C-O, C=O, and COOH) down to < 1–1.2 at.% surface concentration was determined by XPS analysis. The I_D_/I_G_ ratio remained however quite high: 0.83. 

**Figure 7:**
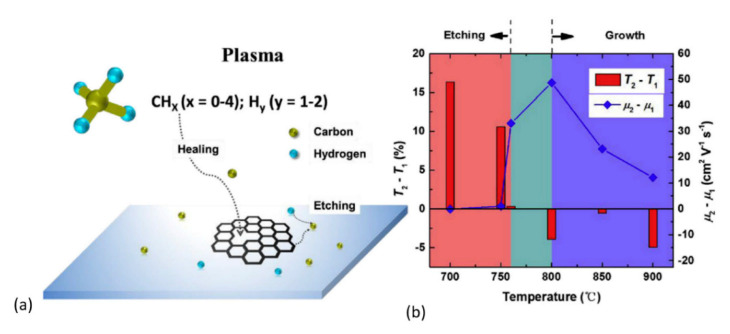
(**a**) Schematic representation of rGO recovery in a methane plasma. (**b**) Variation in the rGO film optical transmittance (at 550 nm) and charge mobility before (T_1_ and μ_1_) and after (T_2_ and μ_2_) a 1-min methane + hydrogen plasma treatment from 700 to 900 °C. The regions of T_2_ − T_1_ > 0 and T_2_ − T_1_ < 0 are denoted as “etching” and “growth”, respectively. Reprinted from Zhu et al. [179], Copyright © 2017, with permission from Elsevier.

**Figure 8:**
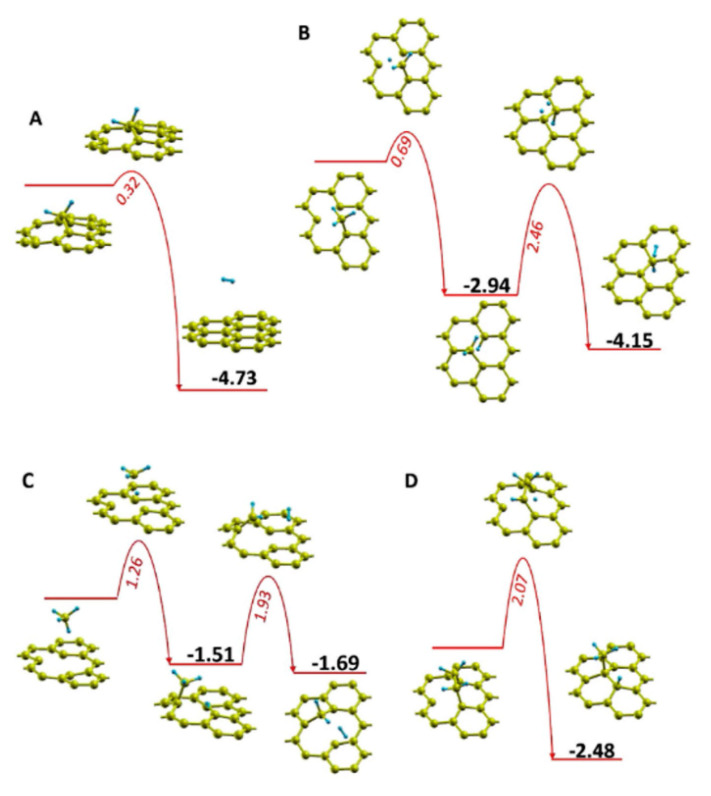
Migration path and its associated energy change for repairing the graphene with mono-vacancy after reaction with (**A**) CH_2_, (**B**) CH_3_, (**C**) CH_4_, and (**D**) CH_2_CH_2_. The red and cyan balls represent the carbon and hydrogen atoms. All energy values are in eV. Reprinted from Zhu et al. [179], Copyright © 2017, with permission from Elsevier.

The work of Zhu et al. [179] sheds more light on the influence of temperature in defect healing and its mechanism. Figure 7a presents a schematic where the CH_x_ (x < 4) species (from dissociated CH_4_) and H_y_ (y < 2) species (from dissociated H_2_) are simultaneously involved in the healing and etching process in GO, respectively. As one can see in Figure 7b, the etching by hydrogen plasma interchanges with growing by methane plasma at ~760 °C. After plasma treatment at 800 °C for 40 s, nearly graphene-like films were obtained from GO-sheets. Additionally, Zhu et al. [179] performed density functional theory (DFT) calculations to elucidate different repairing events during thermal annealing in the plasma environment. Migration paths and their associated energy barriers for repairing a carbon mono-vacancy were presented for various derivatives of dissociated CH_4_ molecule (see Figure 8). As shown in Figure 8, the CH_2_ species possessed a relatively small energy barrier (0.32 eV) compared to CH_3_ (0.69 eV), CH_4_ (1.26 eV), and C_2_H_4_ (2.07 eV) species, indicating that the former species dominate in the repair of the carbon mono-vacancies. The authors also suggested that a CH radical could directly fill a mono-vacancy, but a hydrogen atom should simultaneously be taken away by another radical. To our knowledge, there are no other fundamental reports discussing plasma-assisted reduction and repair of GO yet. However, conventional thermal and chemical reduction methods have been relatively well studied and understood [126,210,222,223,224,225]. 

An interesting question to be understood is whether the reduction and defect healing can be extended to the multi-layered GO, namely, deeper layers that are not directly exposed to plasma. Obata et al. [184,189] demonstrated RF CH_4_/Ar plasma (10 W, 550 °C) reduced GO-monolayers with electron mobility of 460–900 cm^2^/V·s and contributed detailed experimental investigations. On the downside, the process needed a remote Cu-film in the vicinity of GO (up to 8 mm distance) to catalyze the reduction and restoration. The authors characterized the plasma effect on mono-, bi-, and tri-layered GO by Raman spectroscopy [184]. As the number of layers increased, the spectra deviated from graphene-like ones and resembled like rGO (larger I_D_/I_G_, smaller I_2D_/I_G_, wider G-band). This indicated that while the topmost surface changed to restored graphene the deeper layers changed to rGO only. The latter transformation was supposedly driven by the heat of plasma, while the penetration of the dissociated CH_4_ species was apparently restricted to the top surface layer. Obata et al. [184] attributed this to the selective permeability of graphene and GO, which is in turn dependent on the kinetic diameters of the involved species [226,227]. The defect repair by plasma method thus has a limitation to GO-monolayers [48].

### 3.4. Nitrogen and Ammonia Plasma

**Figure 9:**
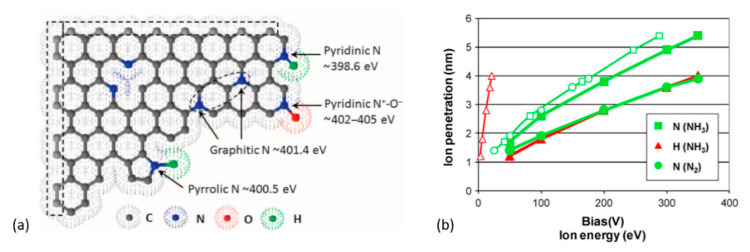
(**a**) Illustration of commonly doped nitrogen species in graphitic carbon with the corresponding XPS binding energies. Reprinted from Zhang et al. [253]. © The Authors, some rights reserved; exclusive licensee AAAS. Distributed under a Creative Commons Attribution NonCommercial License 4.0 (CC BY-NC). (**b**) The penetration depth of N and H ions into graphene oxide produced from NH_3_ and N_2_, calculated as a function of the applied bias (filled marks) and ion energy (open marks). Reproduced with permission from Mohai et al. [181]. Copyright © 2018 John Wiley & Sons, Ltd.

Using the N_2_ or NH_3_ plasma treatment is a way to dope graphene by nitrogen [14,228,229]. Amongst various doping techniques available, the plasma-assisted methods yield higher N-atomic concentrations [230]. In the case of GO, plasma treatment not only results in N-doping, but also provokes simultaneous deoxygenation/reduction. The N-atom incorporated into the graphitic network may be pyridine-like, pyrrole-like, or graphite-/quaternary-like (see Figure 9a) [231,232]. The doping of graphene and GO by heteroatoms (boron, nitrogen, phosphorus, sulfur, halogens, etc.) was thoroughly discussed by Wang et al. [233]. Briefly, the quaternary-N defects donate to the graphene lattice ~0.5 free electrons per site [234,235]. In contrast, the pyridinic-N and pyrrolic-N are acceptor defects [235]. However, on hydrogenation, the pyridinic-N doping transforms from *p*- to *n*-type [235]. The pyridinic-N and pyrrolic-N sites were suggested to promote electrocatalytic properties [236,237]. N-doped GO materials also have potential for use in fuel-cells [238], water-splitting [239], batteries [240], supercapacitors [241,242], and perovskite solar cells [243].

The dissociation energy of N_2_ is higher than NH_3_; therefore, N_2_ plasma ensures less density of active species than a NH_3_ plasma for the same given conditions [244]. Additionally, a N_2_ gas discharge constitutes excited species such as N, N^+^, N_2_^+^, etc. [245], whereas an NH_3_ plasma can constitute H, N, N_2_, NH, NH_2_, N_2_H_2_, N_2_H, NH_2_^+^, NH_3_^+^, and NH_4_^+^ excited species [246,247,248,249]. Well-known reducing agent hydrazine (N_2_H_4_) has also been observed at certain conditions in NH_3_ plasma [250,251]. Thus, more active and reductive NH_3_ plasma should be more effective than a pure N_2_ plasma. Charged radicals N_x_H_y_ (from dissociated NH_3_, mostly N_2_H radicals) were suggested to reduce isolated epoxide groups in GO [252], while -NH_2_ groups were suggested to substitute hydroxyl groups [210]. Mohai et al. [181] subjected GO-films to N_2_ and NH_3_ plasma, biasing the sample’s potential to accelerate ions towards the GO-surface. The authors found by XPS that the NH_3_ plasma effectively reduced the amount of oxygen on the GO surface. The penetration depth of bombarding ions was also computed in this work that revealed the following: (a) biased substrate potential makes penetration depth of N-ions greater than that of H-ions; (b) N-atoms from NH_3_ could penetrate deeper than those from N_2_ for a given biasing energy (see Figure 9b).

**Figure 10:**
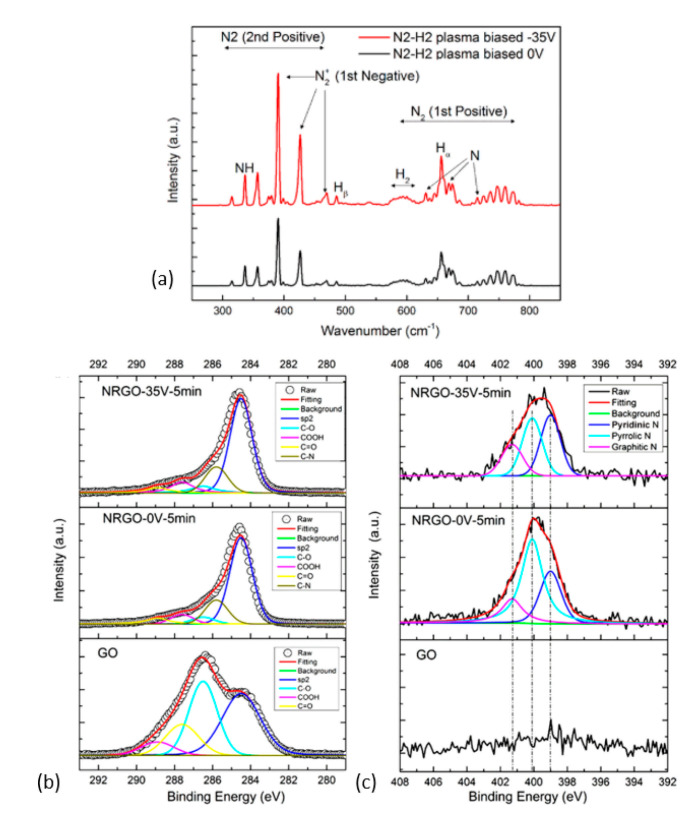
(**a**) Optical emission lines from 200 to 800 nm of N_2_ and H_2_ plasma with 1400 W input power at the pressure of 1.6 Pa (ICP, RF—370 kHz). XPS C1s spectra (**b**) and N 1s spectra of GO (**c**) of reduced GO samples without bias (N-RGO-0V-5min) and with a −35 V bias (N-RGO-35V-5 min). Reprinted with permission from Zhou et al. [187]. Copyright © 2019 American Chemical Society.

The use of an N_2_ plasma can be effective when H_2_ gas is admixed. Zhou et al. [187] used this combination (N_2_/H_2_: 40/10 sccm, 1400 W, ICP (~370 kHz)) along with negative-voltage sample biasing (−35 V) to direct and accelerate the positive ions towards the sample surface. The OES data (Figure 10a) confirmed the existence of several radicals from the dissociation of N_2_ and H_2_ molecules where the N_2_^+^ species dominated. The electron temperature (T_e_) in plasma increased from 0.55 eV when no bias voltage was used to 0.79 eV in the opposite case. The increase in T_e_ in turn activated more N_2_ molecules. The •NH radical was attributed to the removal of isolated epoxide groups, while the reactive H-ions was attributed to removal of hydroxyl groups. Using bias voltage also increased the share of graphitic-N sites from 16.88% to 21.11% and pyridinic-N from 27.55% to 38.70% (Figure 10b,c) in the total N-content.

The share of pyridinic, pyrrolic, and graphitic sites in the N-doped rGO varies from report to report based on experimental conditions. A majority of the considered reviews reported that the share of pyrrolic-N was the maximum according to XPS [187,192,195,197,254]. Graphitic-N possessed a high share when processed at higher temperatures [186]. There has been an instance where the graphitic-N share was the highest even after treatment at room temperature [198]. Calculation of formation energies suggested that the substitutional N-doping (graphitic-N) is the most favorable among other configurations [255]. In a presence of carbon vacancy, however, the pyridine-N becomes energetically favorable. Another study pointed out the preference of graphitic-N to appear at carbon atoms near other defects [256]. This study also concluded that pyridine- and pyrrolic-like configurations mostly realized on mono- and di-vacancies. The N-configurations were observed to vary in graphene depending on N_2_^+^-ion beam energy and irradiation time [257]. Similarly, sputtering GO with 220 eV N_2_^+-^ions for a variable time resulted in different N-configurations and deoxygenation extents [258]. More research must be done in this context for applications where specific N-configuration on GO needs to be tailored. 

Lee et al. [197] carried out reduction and nitridation of the 10 nm thick GO-films in the bulk and sheath regions of an NH_3_ RF plasma (1000 W/m^2^ power density). The sheath region is characterized by a much stronger electric field than in the glow discharge region that provides domination of ion bombardment [197]. Chemical reactions dominate in-turn in the glowing volume. The XPS investigation revealed that even when placed in a sheath, the GO sample partially lost oxygen and gathered nitrogen, but not as effectively as when treated in a chemically active glow. The samples placed in the sheath were not conductive, which was expected to result from ion bombardment breaking the graphitic network. The authors proposed the following reactions by radicals or ions to be responsible for the reduction of GO in a glowing region of plasma:

NH_2_ + H + C_g_-O-C_g_ → C_g_-NH_2_ + C_g_-OH (1)

NH + H_2_ + C_g_-O-C_g_ → C_g_-NH-C_g_ + H_2_O (2)

NH_2_ + H + C_g_-OH → C_g_-NH_2_ + H_2_O (3)

NH_2_ + H + C_g_-COOH → C_g_-CO-NH_2_ + H_2_O (4)

NH_2_ + H + C_g_-COOH → C_g_-NH_2_ + H_2_O + CO + H_2_O (5)

where C_g_ is a carbon atom on the regular graphene site. Equations (1) and (2) explain the reduction of epoxide groups, Equation (3) corresponds to a reduction of the hydroxyl group, and Equations (4) and (5) correspond to that of carboxyl groups on the GO-film. 

### 3.5. Acetylene Plasma

An acetylene discharge is also a source of carbon species assisting the healing of GO. To our knowledge, there are only two reports on this topic [186,259]. In both cases, the GO films were annealed in a vacuum (up to 6 h long) before plasma treatment. Such pretreatment could have caused deoxygenation and formation of carbon vacancies, which are active sites for repair. In one of those works, Ooi et al. [259] successfully replaced indium–tin–-oxide (ITO) with C_2_H_2_/H_2_ plasma reduced rGO for an application in a memory device. Before plasma treatment (20 W, 700 °C, 2 min), the GO was thermally annealed at 700 °C for 6 h. Thus, the obtained ~5.12 nm thick rGO film exhibited ~90 cm^2^/V·s and sheet resistance of ~510 Ω/sq. From the reported thickness and sheet resistance, we estimate the conductivity to be ~3830 S/cm, which is one of the highest reported values for rGO. 

Similarly, Haniff et al. [186] prepared rGO on eight-inch Si/SiO_2_ wafer using C_2_H_2_/NH_3_ plasma (20 W, 700 °C, 3 min) for photodetector application. Before plasma treatment, the films were also annealed in a vacuum (700 °C, 30 min). Since NH_3_ was added to the discharge gas, the films became N-doped. From the point of the mechanism, it was suggested that active hydrogen species react with oxygen groups at first and eliminate them creating active sites (e.g., point defects and vacancies) for the following N-doping. Simultaneously, the dissociated C_x_H_x_ (x < 2) and NH_x_ (x < 3) radicals interact with active sites restoring graphitic plane with C- and N-atoms. The photodetectors obtained in this work revealed a photoresponsivity of 0.68 AW^−1^ at 1.0 V, outperforming graphene-based photoconductor by ~2 orders of magnitude. Although impressive applications have been demonstrated, acetylene (and other carbonaceous gases) has not been studied to the same extent as methane for plasma-assisted defect healing in GO. Theoretical and experimental research is needed to identify which mechanisms and carbonaceous gas discharge are chemically advantageous for low-temperature defect healing. Gong et al. [260] have investigated the defect repair of GO in the presence of water, methanol, and ethanol and concluded that ethanol can contribute better to reduction with increased defect repair in GO. 

### 3.6. Air Plasma

**Figure 11:**
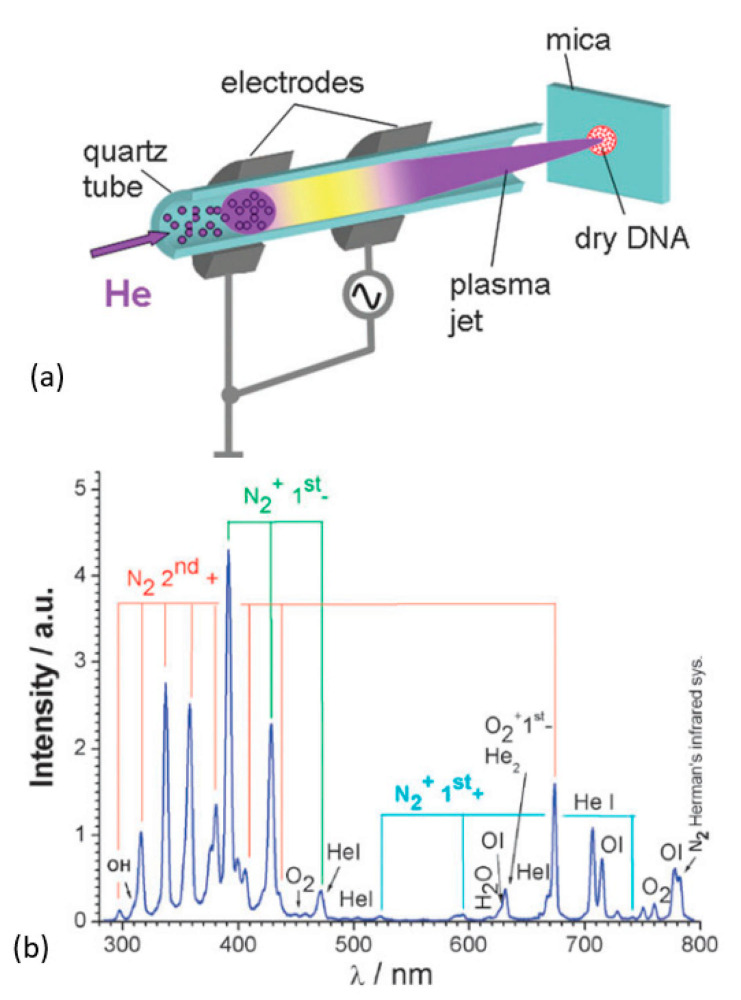
(**a**) Schematic of an APPJ-plasma (20 W, RF) ignited with He (10 m/s) and launched through ambient air on to the sample. (**b**) The optical emission spectra of the corresponding plasma. Reproduced from Ptasinska et al. [269] with permission from the Royal Society of Chemistry.

GO-reduction using air as the discharge gas in APPJ-plasma has been demonstrated [205,261]. The APPJ-plasma is created either with a noble gas or air, and the reactive species are generated within the plasma (e.g., He + O_2_ admixture) or its interaction with the surrounding (air: mostly N_2_, O_2_, and H_2_O vapor) [262]. Thus, various atoms, radicals, ions, and excited molecular species such as: O, O_3_, ^1^O_2,_ •OH, N_2_, N_2_^+^, O_2_^•−^/^•^OOH, ^•^NO, ONOO^−^, OONOO^−^, H_2_O_2_, NO_2_^−^, and NO_3_^−^ are produced [262,263,264] (an example, Figure 11). In an air plasma, active oxygen species stipulate its highly oxidative impact, which is used to remove ad- and chemisorbed organics from the treated surface [265,266,267]. 

An RF air-plasma (7 W, 49 mL/min, 30 s) has been used to eliminate polymer residues from the surface of transferred CVD-graphene without damaging the graphitic network [268]. Huang et al. [261] treated the surface of the CVD-graphene monolayer with an APPJ-He-plasma (10.7 kV and 14 mA at the electrode, 100 μs short pulses at 1.5 kHz frequency) in ambient air. They observed that as the treatment time progressed from 0–60 s, the concentration of oxygen-containing groups increased from ~10% to ~40%. At longer treatment (>60 s), GO (oxidized CVD-graphene) started losing oxygen together with carbon. The authors suggested that the epoxide groups are unstable under APPJ conditions due to the intensive bombardment by electrons. At C-O-C dissociation, highly reactive radicals like (C-O•) are formed. After activation of epoxide, the transient surface atomic oxygen is captured by surrounding oxygen, forming a di-oxygen molecule as product. The authors suggested that for graphene with high C-O-C coverage, the rate of reduction exceeded that of oxidation.

Alotaibi et al. [205] performed a room-temperature reduction process on GO-films with a scanning atmospheric plasma. Air was used as discharge gas, and a high potential of ~10 kV was applied between the electrodes (300 W, 20 kHz power supply) to generate an arc discharge. The reduced GO realized by the APPJ process was demonstrated in a supercapacitor with a volumetric capacity of 536.55 F/cm^3^ at a current density of 1 A/g. By combining the plasma jet with software-controlled scanning devices, they were able to demonstrate rapid and controlled GO reduction on several substrates such as glass, plastic, and textile with various shapes and size patterns. 

According to the authors, negatively charged oxygen ions participate in bond-breaking, displacement of atoms on the surface, and charge accumulation. Through inelastic collisions, the ions with low kinetic energy bombard the GO-surface, causing rupture of oxygen-containing groups and release of oxygen ions. As a result, the ions O^2−^ and O_2_^−^ transfer free electrons to the GO-surface and charge it. The formation of nitric oxide (NO) in plasma was also suggested. It can take part in reaction with such groups as -COOH, -CO-, and -OH, converting itself into NO_2_. On a free-standing 25 μm thick GO film, the reduction process in question produced the exposed to plasma rGO surface with sheet-resistance of 186 Ω/sq in case of 1 min treatment and 160 Ω/sq in case of 2 min treatment. The back rGO side also revealed some reduction extent: R_sh_ = 680 Ω/sq (1 min) and 560 Ω/sq (2 min), indicating the penetration effect of plasma. To exclude the heating effect of the plasma beam, the authors performed a control experiment by annealing GO in a convection oven for 2–20 min. Fourier transformed infrared spectroscopy analysis revealed that a min of 20-min thermal annealing was required to achieve a comparable reduction degree with a 10 s plasma reduction. Thus, a major impact of plasma by heating was ruled out. 

## 4. Summary and Conclusions

The plasma environment with free-electrons and reactive species reduces GO. In the case of a controlled potential difference between plasma and GO surface, energetic ions bombard the surface, distorting and breaking various chemical bonds. Oxygen-containing groups escape upon further bombardment of plasma species that finally results in GO reduction. Plasma, especially in its equilibrium state, and plasma irradiation generate heat in a treated solid. In that sense, the mechanism of GO reduction by plasma involves dynamic events typical for established thermal reduction. However, plasma processes offer additional parameters to tune: pressure and chemistry of the discharge gas, type and frequency of power generation, and exact treatment time. The set of different types of species (ions, excited atoms or molecules, and electrons) and their relative energy and chemical activity defines the penetration depth and the chemical impact. 

Within the scope of reviewed literature here, three main missions of plasma reduction can be distinguished: (I) reduction for improved electrical conductivity (and higher R_C/O_ than initially), which is the general case, (II) reduction with simultaneous doping (mostly by nitrogen), and (III) restoration of graphitic network with mostly sp^2^-carbon hybridization that means healing of lattice defects. In the general case, all plasma treatments, i.e., based on inert gases (Ar and He) or reactive ones (H_2_, N_2_, NH_3_, CH_4_, and C_2_H_2_) or a combination of them, serve the purpose of reduction. For the second task, plasma needs to contain dopant species (dissociated N_2_ or NH_3_) for insertion into the carbon lattice along with reduction. The third task requires the presence of carbon species in plasma for assisting in the healing of GO-defects and increasing sp^2^-carbon domain size. The corresponding discharge processes are based on a carbonaceous gas (like CH_4_ and C_2_H_2_) and moderate- to high-substrate temperature (230–800 °C). The defect restoration is however limited to monolayers that are nonetheless attractive for the realization of graphene-like electrical properties. Using this approach has already demonstrated conductivities in the order of ~10^3^ (S/cm) and electron mobility ~900 cm^2^/V.s. To compare, a monolayer film of CVD-graphene has electrical conductivity in the order of 10^4^ S/cm [270,271] and an R_C/O_ of 20 [272]. Certain plasma reduced rGO have attained conductivities in the same magnitude as that of certain highest reported for other techniques such as chemical assisted thermal annealing (acetylene, 1000 °C, 2 h, 1425 S/cm) [273], joule-heating (>2000 K, 1500 S/cm) [274], and two-step treatments: thermal annealing (Ar, 1000 K, 1 h) and joule heating (3000 K, 6300 S/cm), and chemical reduction (hydrazine monohydrate) and graphitization (2700 °C, 5770 S/cm) [220]. These techniques compared to the plasma methods are inferior for large-scale applications. In the case of reduction at a lower temperature regime, which is particularly interesting for flexible applications, the rGO obtained from the plasma method has demonstrated to be superior to those obtained from the thermal method and is comparable to ones obtained from the chemical method [56,162,202]. 

In certain cases, a mixture of gases can enhance the reduction efficiency of plasma. For instance, in a mixture of inert and reactive gases, active species of the former enhance the dissociation degree of molecules of the reactive gas through penning ionization. Another example: the dissociated hydrogen species (from hydrogen) carry out etching of GO, while the carbon-containing active species (from methane) perform carbon deposition. To optimize such plasma processes, it is crucial to find the right combination of the discharge regime and the ratio of constituent gases for the best cooperative effect. Finally, it is challenging to formulate a single optimal strategy for plasma-assisted reduction of GO just based on the reviewed literature. This arises from the variations (thickness, initial R_C/O_, the density of defects) of GO-precursors used, different plasma instrumentation and conditions, diversity of measurements, etc. Nevertheless, we collected in this work the factual material, which is crucial for understanding the principles of plasma reduction of graphene oxide.

## 5. Outlook

As Eigler et al. [124] pointed out, the quality or defectiveness of precursor-GO material influenced by the preparation conditions limits the potential of reduction. The use of large-sized (>10 μm) GO flakes is beneficial over smaller ones due to reduced defect density and improved electrical, thermal, and mechanical properties [54,275]. Additionally, theoretical studies have shown that the proportion between hydroxyl- and epoxide-groups and the total oxygen coverage of initial GO affect the outcome of a reduction process [222,276]. Therefore, improved control of the state-of-the-art GO syntheses and further processing techniques will certainly improve the effectiveness of the subsequent plasma reduction process. Independently, a basic understanding of the reaction mechanisms in plasma and therefore control of its energetic and chemical footprint will allow mastering the GO reduction. This is valid and even more essential for the simultaneous reduction and doping, as well as for the defect healing with sp^2^-carbon restoration.

A vast majority of the reports are based on low-pressure plasma processes. Breakthroughs with low-temperature and atmospheric-pressure plasma for the same purpose will not just present an opportunity to lower associated costs but importantly facilitate integration with roll-to-roll processing. Low-temperature plasma reduction also will enable novel and niche applications (e.g., in monolithic CIGSe–Perovskite tandem solar cell, as mentioned earlier). Graphene materials are emerging in commercial products, breaking their confinement to academic and research prototypes [277]. Among the graphene-related materials, GO has its significance. According to a 2019 report [278], GO occupies 38% of the market fraction (graphene nanoflakes—52%; CVD-films 10%) with the global production capacity (~784 tons/year in 2017) displaying an increasing trend over the past decade. The global reserve of recoverable graphite exceeds 800 million metric tons as per the 2020 U.S. geological survey [279]. The abundance of raw material reserves along with low-cost and scalable production is encouraging to explore GO/rGO based applications. The amount of scientific research dedicated to plasma technologies has been tremendous over the past decades. However, plasma for GO-reduction has not gained as much substantial attention as the chemical and thermal methods. In today’s material processing world, plasma technology is at the forefront. If backed by persistent research, it is a strong contender for facilitating large-scale applications of rGO. 

## Figures and Tables

**Table 1:** Summary of physical properties of monolayer CVD-graphene, various-GO, and various-rGO. Properties listed are atomic C/O ratio (R_C/O_), optical transmittance (T) at 550 nm for monolayers, electrical conductivity (σ), bandgap (E_g_), thermal conductivity (κ) at room-temperature, in-plane Young’s modulus (E), and intrinsic strength (τ_c_).

Property	CVD-Graphene	Various-GO	Various-rGO
R_C/O_	-	0.6–2.38[47,83,84]	1.48–12 [52,85,86] *
T (%, @ 550 nm)	97.7 [87]	>97.5 [88]	~97.5 [88,89]
σ (S/cm)	~10^4^ [90,91]	~10^−3^–10^3^ [92,93] ^†^	
E_g_ (eV)	0	0–3.5 [94,95] ^†^	
κ (W/m∙K)	300–5300 [96,97]	8.8–625 [98,99] ^†,‡^	46–2600 [100,101]
E (GPa)	1000 [102]	290–430 [103] ^†,‡^	
τ_c_ (GPa)	130 [102]	28–48 [103] ^†,‡^	

* a wide range of R_C/O_ have been reported for rGO [51], in certain cases exceeding >100 [104,105]. ^†^ range obtained as a function of oxidation/reduction degree. ^‡^ theoretical studies.

**Table 2:** A summary of some of the properties and features of GO/rGO with their relevant applications.

Features/Properties	Applications/Technologies	Reference
Large specific area; lightweight; high conductivity; hetero-atom doping; micro-structuring; composite material formation	Electrochemical storage (batteries and capacitors)	[16,114]
Large specific area; tunable electronic structure; hetero-atom doping; structural modification and functionalization	Electrocatalysts for electrochemical energy conversion reactions (water splitting; CO_2_, N_2_, and O_2_ reduction reaction)	[115]
Nanocapillaries; ease of making atomically thin layers; good mechanical properties	Membranes (selective ion-, vapor-, gas-, water-transport; proton exchange; desalination)	[116]
Biocompatibility; functionalization; physiochemical properties; fluorescence	Pharmaceutical, biomedical, and biosensing	[106,117]
Tunable electronic properties; optical transparency; mechanical flexibility	Flexible-, thin-film, and opto-electronics	[106,118]
Non-linear optics (saturable absorption; reverse saturable absorption; two-photon absorption)	Mode-locking; Q-switching; optical limiters	[106]
Seebeck coefficient; electrical conductivity; thermal conductivity	Thermoelectric devices	[106]
Advanced mechanical and structural properties in composites	Mechanical and rheological (cement composites; green plastics; composites for military and aerospace)	[118]

**Table 3:** An overview of the common reduction methods with some of their important features.

Reduction Method	Features	Reference
Chemical	simple and scalable approach; commonly used reducing agents are toxic/hazardous; rGO yields have lower surface area and electrical conductivity; prolonged reduction duration	[94,125]
Thermal	simple approach; defects are created in the lattice with the removal of carbon; high-temperature process not for suitable sensitive substrates; substantial energy consumption	[125,126]
Electrochemical	rGO yields have good structural quality and electrical conductivity; non-hazardous process, large-scale production is challenging	[125]
Microwave-assisted	microwave absorption depends on the oxidation degree of GO; reducing atmosphere are needed to improve quality of yield; high temperatures attained limit substrate selection	[50,127,128,129]
Plasma	requires special equipment; versatile and offers industrial-level scalability; relatively short reaction period; effective in restoration of lattice defects	[49,93,130]

**Table 4:** Characteristics of various plasma sources.

Plasma Source	Breakdown Voltage (kV) [63]	Plasma Density (cm^−3^) [63]	Electron Temperature (eV) ^†^
Low-pressure discharge	0.2–0.8	10^8^–10^13^	0.1–10 [172]
Arc and plasma torch	10–50	10^16^–10^19^	2–7 [63]
Corona	10–50	10^9^–10^13^	5 **^‡^** [61]
DBD	5–25	10^12^–10^15^	1–10 [61]
Plasma jet	0.05–0.2	10^11^–10^12^	1–2 [61]

^†^ 1 eV ≈ 11,604 K;^**‡**^ variable.

## Abbreviations

2D	two dimensional
σ	conductivity
μ-APPJ	micro atmospheric pressure plasma jet
A	ampere
AC	alternating current
AGD	atmospheric-pressure glow discharge plasma
APPJ	atmospheric pressure plasma jet
AS	active screen
CCP	capacitively coupled plasma
CIGSe	Cu(In, Ga)(Se, S)_2_ solar cell
CVD	chemical vapor deposition
DBD	dielectric barrier discharge
DC	direct current
FET	field effect transistor
GO	graphene oxide
hr	hour
ICP	inductively coupled plasma
kΩ	kilo-Ohm
min	minute
mTorr	milli-Torr
MΩ	mega-Ohm
MW	microwave
RF	radio frequency
rGO	reduced graphene oxide
s	second
sq	square
V	volt
V/V	volume/volume
W	watt

## Appendix A

**Table A1:** Overview of various plasma used for the reduction of GO. Temperature in the “Parameters” column denotes auxiliary heated substrate temperature, when a temperature is not specified no external heating was used. In the “Characteristic changes” column, time indicated in brackets correspond to the reduction duration.

Serial No.	Year & Reference	Plasma				Characteristic Changes	Application
		Discharge Gas	Generation	Time	Parameters		
1	2007 [150]	H_2_	--	5 s	30 W, 0.8 mbar	σ: 0.05–2 S/cm, ambipolar charge transport	FET
2	2010 [203]	10% CH_4_ + Ar	Electron beam	30 s	−2 kV pulse at linear hollow cathode with 150 G Helmholtz coil, 90 mTorr	Controlled reduction with atomic oxygen varied between 43% and 5%	--
3	2011 [188]	Ar	RF	5–40 min	25 W, 0.02 mbar, 20 sccm	>2 orders lower surface resistivity. Etching effect with prolonged duration	--
4	2012 [93]	CH_4_	RF	10 min	100 W, 0.20 Torr, ~575 °C	σ: 1590 S/cm, I_D_/I_G_: ~0.53	--
5	2012 [202]	Ar/H_2_ (1:1)	DC	30 min	~2 kV, 10 mA discharge current, atm. pressure, 150 °C	Sheet resistance 47.7 kΩ/sq (~6 μm), C/O ratio 6.95	--
6	2013 [192]	NH_3_	RF	3 min	200W, 500 mTorr, 150 °C, 400 sccm	~6% N-doped r-GO, σ: 7.4 S/cm, C/O ratio ~9.4 I_D_/I_G_: 0.98 (GO:1.09)	--
7	2013 [193]	H_2_	RF	<30 s	200W, 20 mTorr, 150 °C, 300 sccm	σ: 2 S/cm (18 s) and C/O ratio of ~14 (30 s), >18 s exposure causes trade-off between σ & reduction	--
8	2013 [280]	H_2_	--	<10 min	2 W, 1 Torr	Reduction in sheet resistance by 3~4 orders of magnitude that could be restricted to uppermost layers on short exposure	FET
9	2013 [198]	NH_3_	DC	1–20 min	10–30 W, ~1 Pa	C/O ratio: ~ 6.66, N/C ratio: 15%, work-function change from 4.4 to 3.4 eV, σ: 1–80 S/cm (5 min)	--
10	2013 [191]	(a) NH_3_ (b) H_2_ + 10% Ar	-	(a) 4.5–10 min (b) 8.5 min	160 W, 8 sccm	(a) μ_e_: 5.41 cm^2^/V.s (8.5 min) and μ_h_: 2.1 cm^2^/V.s (5.5 min) (b) σ: 630 S/cm for (8.5 min)	FET
11	2014 [194]	H_2_	RF	40 s	10 W, 0.3 mbar, 20 sccm	C/O ratio ~7.9, I_D_/I_G_: 0.81 (GO: 0.94), 71% (15%) response at 1500 ppm in N_2_ (in air)	CO_2_ gas-sensor
12	2014 [199]	H_2_	DC	10 s–5 min	15 & 30 W, working pressure ~50 Pa, 50–120 °C	σ: 0.2–31 S/cm & μ_h_: 0.1–6 cm^2^/V.s (15 W, 50 °C, 30 s)	FET
13	2014 [195]	NH_3_	RF	30 min	10 W, 100 mTorr	σ: 1666 S/m, C/O ratio ~4.16 and N/C ratio 9.3%, I_D_/I_G_: 1.84 (GO: 2.22)	--
14	2014 [56]	He	AGD	2 s	−10 kV, discharge current ~1.5–1.9 mA, atmospheric pressure (positive-column plasma)	σ: 5900 S/m, C/O ratio 7.6, surface area: 371 m^2^/g, specific capacitance 161.6 F/g (1 A/g)	Supercapacitor
15	2014 [190]	H_2_/Ar (various ratio)	RF	3–10 min	20–100 W, 4.7 Pa	C/O ratio: 9.6 (100 W, H_2_/Ar-2:1, 5 min), specific capacitance 185.2 F/g (100 mV/s) for 70 W, H_2_/Ar-2:1, 5min (C/O ratio 4.2)	Supercapacitor
16	2015 [200]	Ar	DC	4 min	~10 kV at cathode, 240 mTorr	C/O ratio ~7.9, I_D_/I_G_: 0.85, specific capacitance of 190 F/g (10 mV/s)	Supercapacitor
17	2015 [196]	H_2_	RF	30 min	60 W	σ: 3.1 S/cm, μ: 37.5 cm^2^/V.s	--
18	2015 [204]	25% N_2_ + H_2_	AS	60 min	4 mbar in a traditional 40 kW plasma nitriding unit, 100–200 °C	Doping of N from gas discharge and Fe, Cr, and Mo elements from steel mesh, reduction in resistance from 12.6 MΩ to 50 kΩ (200 °C)	--
19	2015 [197]	NH_3_	RF	1–40 min	1 kW/m^2^_,_ 500 mTorr	C/O ratio ~2.7 and N/C ratio ~17% (30 min), σ: 80 S/m (30 min)	--
20	2016 [221]	H_2_ + CH_4_	RF	5–30 min	100 W, 240 °C	All oxygen containing groups were removed except ~1.2%, I_D_/I_G_: 0.83, R_SH_: 15 k Ω/sq	--
21	2016 [178]	(a) Ar (b) N_2_	RF	10 min	100 W, 0.3 Pa, sample biased at 0 V and sample bias from −50 to −300 V	N_2_-plasma was more effective in reduction (at all sample bias) with relatively higher ion penetration depth	--
22	2017 [207]	H_2_, CH_4_, & H_2/_CH_4_ (1:1)	DBD	1–10 s	1.9 W/cm^2^, each gas-flow at 0.2 mL/min	~40% sp^2^—Carbon restored. Oxidation level in GO reduced from ~50% to ~10% (H_2/_CH_4_)	--
23	2017 [179]	10% CH_4_ + H_2_	RF	~1 min	Thermal annealing (Ar, 1000 °C, 30 min) followed by plasma treatment: 200 W, 0.34 Torr, 700–900 °C	improvement in μ from 0.01–1 cm^2^/V.s to 50 cm^2^/V.s., I_D_/I_G_: 0.56 (GO:1.04)	--
24	2017 [259]	20% H_2_ + C_2_H_2_	-	2 min	Thermal annealing (vacuum, 700 °C, 7 hrs.) followed by plasma treatment: 20 W, 50 sccm total flow	μ_hall_: ~90 cm^2^/V.s, R_SH_: 510 Ω/sq (5 nm), C/O ratio 10.9, I_D_/I_G_: 1.33 (GO:0.97)	Memory device
25	2017 [180]	5% CH_4_ + Ar	RF	1–20 min	700 W/m^2^, 500 mTorr	transmittance from 91.9% to ~94% at 600 nm & 8.13 MΩ/sq (10 min)	--
26	2018 [181]	(a) NH_3_ (b) N_2_	RF	10 min	100 W, 0.3 Pa, sample biased at 0 V and −50 to −350 V	Both plasmas incorporated N into GO, NH_3_ was more effective in reduction of O-groups	--
27	2018 [208]	H_2_	DBD	1–64 s	40 W/cm^3^_,_ atm. pressure	Resistance reduced to 2 MΩ/sq, C/O ratio of ~4.76 (16 s)	--
28	2018 [205]	Air	RF	10–120 s	300 W, 20 kHz, ~10 kV (APPJ plasma)	R_SH_: 186 Ω/sq (25μm), vol. capacitance 536.55 F/cm^3^ (1 A/g)	Supercapacitor
29	2018 [184]	H_2_/CH_4_ (1:1)	RF	10–120 min	10 W, 9.7–9.8 Pa, H_2_ & CH_4_: each at 35 sccm flow, 550–650 °C with Cu catalyst	σ: 930 S/cm, μ_e_: 480 cm^2^/V.s, I_D_/I_G_: ~0.4	FET
30	2018 [183]	H_2_	RF	30 min	20–60 W	Rise in optical bandgap energy with increase in O-vacancy	--
31	2018 [201]	H_2_	DC	1 min	15 W, ~0.3 Torr	Threshold of device reduced from 2.6 to 1.8 V, On/Off ratio improved from ~80 to ~10^3^	Memory device
32	2018 [185]	CH_4/_Ar (1:2 to 2:1)	RF	1–10 min	100 W, 50 mTorr, total gas flow 30 sccm	C/O ratio improved from 2.2 to 10.6 & σ: 264 S/m (5 min)	--
33	2019 [189]	H_2_/CH_4_ (35:1 to 1:35)	RF	~0.16–9 hrs	10 W, 9.7–9.8 Pa, total flow 350 sccm, 550 °C with Cu catalyst	Moderate restored r-GO in ~15 min for CH_4_ rich condition, high crystallinity for H_2_ rich condition: μ_e_: 900 cm^2^/V.s, I_D_/I_G_: 0.17	--
34	2019 [186]	20% C_2_H_2_ + NH_3_	-	3 min	annealing at 700 °C (30 min, 10^−4^ mbar); followed by 3 min plasma: 20 W, 2 mbar, 50 sccm total flow	C/O ratio 8.34, μ_e_: 60–80 cm^2^/V.s, photoresponsivity of 0.68 A/W at 1 V	Photodetector
35	2019 [187]	20% H_2_ + N_2_	RF	5–80 min	1400 W at ~370 kHz, 50 sccm total flow, sample with 0 and −35 V bias	DC bias contributed to efficient reduction and recovery of GO (lowest I_D_/I_G_ ratio). Resistance change between ~425–570 kΩ for 0–1.5% strain	Stress sensor
36	2019 [162]	Ar	RF	30–120 min	300 W, 650 mTorr	resistance of 2 kΩ/sq (120 min), mean sensor sensitivity of 277 ± 80 µA/mM.cm^2^; Pt reference 5.2 ± 0.51 µA/mM.cm^2^	H_2_O_2_ sensor
37	2020 [206]	1% He + N_2_ & He + H_2_	RF	5 s–3 min	4 W, flow: 2000 sccm N_2_ & 16 sccm He (µAPPJ plasma)	Sheet resistance: ~4 MΩ/sq (He + N_2_), ~0.24 MΩ/sq (He + H_2_); GO ~138 MΩ/sq	--
